# Flexible electronics for cardiovascular healthcare monitoring

**DOI:** 10.1016/j.xinn.2023.100485

**Published:** 2023-07-24

**Authors:** Tianqi Zhang, Ning Liu, Jing Xu, Zeye Liu, Yunlei Zhou, Yicheng Yang, Shoujun Li, Yuan Huang, Shan Jiang

**Affiliations:** 1 Hangzhou Institute of Technology, Xidian University, Hangzhou 311200, China; 2 Department of Gastrointestinal Surgery, Hainan General Hospital, Hainan Affiliated Hospital of Hainan Medical University, Haikou 570311, China; 3 State Key Laboratory of Cardiovascular Disease, Fuwai Hospital, National Center for Cardiovascular Diseases, Chinese Academy of Medical Sciences and Peking Union Medical College, Beijing 100037, China; 4 Department of Structural Heart Disease, National Center for Cardiovascular Disease, China & Fuwai Hospital, Chinese Academy of Medical Sciences & Peking Union Medical College, Beijing 100037, China; 5 State Key Laboratory of Cardiovascular Disease, Fuwai Hospital, National Center for Cardiovascular Diseases, Pediatric Cardiac Surgery Center, Fuwai Hospital, Chinese Academy of Medical Sciences, and Peking Union Medical College, Beijing 100037, China

## Abstract

Cardiovascular diseases (CVDs) are one of the most urgent threats to humans worldwide, which are responsible for almost one-third of global mortality. Over the last decade, research on flexible electronics for monitoring and treatment of CVDs has attracted tremendous attention. In contrast to conventional medical instruments in hospitals that are usually bulky, hard to move, monofunctional, and time-consuming, flexible electronics are capable of continuous, noninvasive, real-time, and portable monitoring. Notable progress has been made in this emerging field, and thus a number of significant achievements and concomitant research prospects deserve attention for practical implementation. Here, we comprehensively review the latest progress of flexible electronics for CVDs, focusing on new functions provided by flexible electronics. First, the characteristics of CVDs and flexible electronics and the foundation of their combination are briefly reviewed. Then, four representative applications of flexible electronics for CVDs are elaborated: blood pressure (BP) monitoring, electrocardiogram (ECG) monitoring, echocardiogram monitoring, and direct epicardium monitoring. Their operational principles, progress, merits and demerits, and future efforts are discussed. Finally, the remaining challenges and opportunities for flexible electronics for cardiovascular healthcare are outlined.

## Public summary

• Flexible electronics can revolutionize cardiovascular healthcare monitoring.
• Flexible electronics aid in promptly identifying cardiovascular disease indicators, enabling better personalized treatment.
• Further developments include wireless design, miniaturization, multifunction, artificial intelligence-assisted diagnosis, virtual medicine, customized healthcare, etc.

## Graphical abstract

**Figure:**
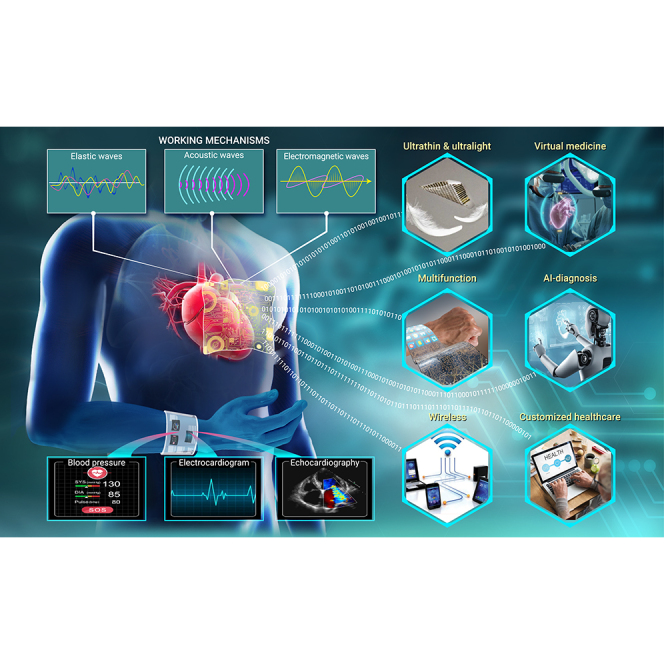

## Introduction

Cardiovascular diseases (CVDs), such as coronary artery disease, stroke, heart failure, hypertension, and arrhythmias, have become the leading cause of death around the world. According to the World Health Organization (WHO), an estimated 17.9 million deaths caused by CVDs occur each year, approximately 31% of all global deaths.^1^^,^^2^^,^^3^ Low- and middle-income countries often face a worse situation. The risk of CVDs increases with age, and they are more commonly observed in individuals over 40 years old. Men have traditionally shown a higher prevalence of CVDs, but the gap has been closing in recent years. As the global aging of the population is becoming increasingly serious, it is foreseeable that deaths from CVDs will continue to increase.^4^^,^^5^ The main causes and risk factors include unhealthy lifestyle, hypertension, high cholesterol, diabetes, and family history. Early detection and intervention and regular risk screening can significantly reduce the incidence of CVDs.^6^^,^^7^ Especially, precise tests of the heart and blood vessels can effectively facilitate treatment of CVDs.^8^^,^^9^^,^^10^^,^^11^^,^^12^ Currently, blood pressure (BP), electrocardiogram (ECG), echocardiogram, and direct epicardium monitoring are the principal ways of screening for CVDs.^13^^,^^14^^,^^15^^,^^16^ Among these, BP is the most basic but important biometric with hypertension being the most significant modifiable risk factor for CVDs.^10^ Continuous ECG monitoring helps detect arrhythmias and associated heart conditions in specific cases.^17^ Echocardiograms provide various types of information about the structure and function of the heart. Direct epicardium monitoring allows more precise and real-time monitoring of the electrical activity and function of the heart (e.g., electrophysiological data, myocardial ischemia, and viability). However, the conventional medical instruments used for these types of monitoring in hospitals are usually bulky, hard to move, monofunctional, and highly dependent on physicians, resulting in time expenditure and unavailability of timely services, particularly in emergencies. Therefore, there is a growing demand for solving such challenges in cardiovascular healthcare. Recently developed flexible electronics have made breakthroughs regarding such limitations and open a new avenue in healthcare approaches.

**Figure 1:**
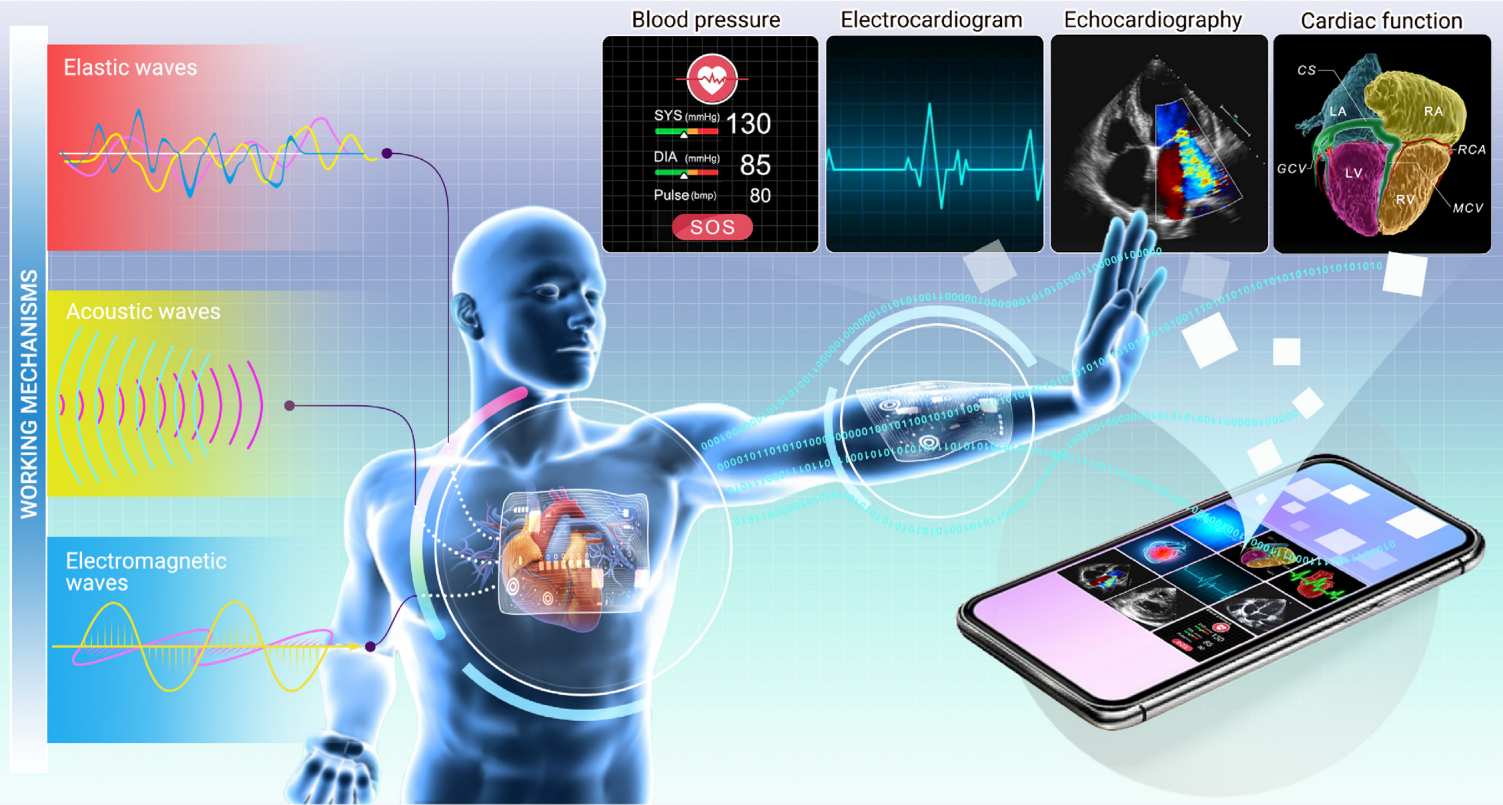
Conceptual illustration of flexible electronics for cardiovascular healthcare monitoring Flexible electronics are an almost ideal platform for customized and personalized healthcare, capable of being operated by patients. They enable continuous, *in situ*, real-time, and comfortable BP, ECG, echocardiogram, and direct epicardium monitoring, offering clinically related indicators for preventive healthcare and disease diagnosis. According to the fundamental mechanisms, flexible electronics for cardiovascular healthcare can be divided into three categories: (1) flexible mechanical electronics, which rely on mechanical deformation and interaction with elastic waves; (2) flexible optical electronics, which exploit light-matter interaction; and (3) flexible acoustic electronics, which utilize sound-matter interaction.

The advent of flexible electronics has brought a novel epoch of comfortable and user-oriented devices. Supple, foldable, bendable, and ultimately conformal, wearable electronics are revolutionizing our lifestyle.^18^^,^^19^^,^^20^^,^^21^ Unlike conventional silicon-based rigid electronics restrained by Moore’s law, flexible electronics are thin, lightweight, low-modulus, and stretchable, rendering them “mechanically invisible” to objects with arbitrary surfaces.^22^^,^^23^^,^^24^^,^^25^ They can be mounted onto skin, coupled to clothing, and even implanted into bodies, *viz.* epidermal electronics,^26^^,^^27^^,^^28^^,^^29^^,^^30^ wearable electronics,^31^^,^^32^^,^^33^^,^^34^^,^^35^^,^^36^^,^^37^^,^^38^ and implantable electronics.^39^^,^^40^^,^^41^^,^^42^^,^^43^^,^^44^^,^^45^ Flexible electronics provide an almost ideal platform for customized and personalized healthcare capable of being operated by patients. They enable noninvasive, continuous, *in situ*, real-time, and comfortable monitoring of key biosignals, providing clinically related indicators for preventive healthcare and disease diagnosis.^46^^,^^47^ Moreover, flexible electronics are particularly beneficial for tracking and monitoring chronic symptoms such as metabolic disorders, cardiovascular problems, and diabetes,^48^ which are of importance in older populations. Here, we focus on application of flexible electronics for cardiovascular healthcare, as shown in Figure 1. With continuous and reliable BP, ECG, echocardiogram, and direct epicardium monitoring,^49^ flexible electronics can improve patient outcomes and facilitate personalized treatment.^50^^,^^51^ Such devices can be mounted to various body parts, such as the arm, wrist, chest, and head, as well as bodily fluids.^52^^,^^53^^,^^54^ According to the fundamental mechanisms, flexible electronics for cardiovascular healthcare can be divided into three categories: (1) flexible mechanical electronics, which rely on mechanical deformation and interaction with elastic waves; (2) flexible optical electronics, which exploit light-matter interaction; and (3) flexible acoustic electronics, which utilize sound-matter interaction.

Because of the promising prospects, research on flexible electronics for cardiovascular healthcare has drawn growing attention in recent years. Many impressive and exciting achievements have been made, for instance, multiplexed flexible silicon transistors for long-term cardiac electrophysiology,^55^ conformal ultrasound devices for central BP waveforms,^56^ epidermal electronic systems for neonatal intensive care,^57^ photonic skin for continuous biosignal detection,^58^ and wearable ultrasound devices for continuous, real-time, and direct cardiac function assessment.^59^ Many reviews from different perspectives have been published. However, the majority of them focus on conceptual innovations or novel functions. A comprehensive overview from a perspective of practical clinical application is needed to advance these types of electronics. In this review, we summarize state-of-the-art advances of flexible electronics for cardiovascular healthcare, focusing on their application in BP, ECG, echocardiogram, and direct epicardium monitoring. We also highlight the development, merits and demerits, and future directions. Finally, our perspectives on the remaining challenges and opportunities are outlined. Table 1 offers an overview of flexible electronics for CVDs and their advantages, classification, and corresponding figures and sections in this work.

**Table 1:** Flexible electronics for cardiovascular healthcare monitoring and their advantages, classification, figures, and sections

Objects	CVDs	Flexible electronics	Classification		Figure
Pulse and BP monitoring (Section flexible electronics for pulse and BP monitoring)	hypertension, stroke, diabetes	comfortable, noninvasive, continuous, *in situ*, real time, easy to operate, biocompatible, biodegradable potential ideal platform for customized and personalized healthcare capable of being operated by patients	flexible mechanical sensors (Subsection flexible mechanical sensors for pulse and BP monitoring)		Figure 2
			flexible optical sensors (Subsection flexible optical sensors for pulse and BP monitoring)		Figure 3
			flexible acoustic sensors (Subsection flexible acoustic sensors for pulse and BP monitoring)		Figure 4
ECG monitoring (Section flexible electronics for ECG monitoring)	arrhythmia, myocardial infarctions		epidermal electrodes (Subsection epidermal electrodes for ECG monitoring)	wet electrodes	Figure 5
				dry electrodes	Figure 6
			implantable electrodes (implantable electrodes for ECG monitoring)		Figure 7
Echocardiogram monitoring (Section flexible electronics for echocardiogram monitoring)	myocardial dilatation, pericardial effusion		flexible acoustic imagers		Figure 8
Direct epicardium monitoring (Section flexible electronics for direct epicardium monitoring)	myocardial infarction, cardiomyopathy		flexible mechanical sensors		Figure 9

**Figure 2:**
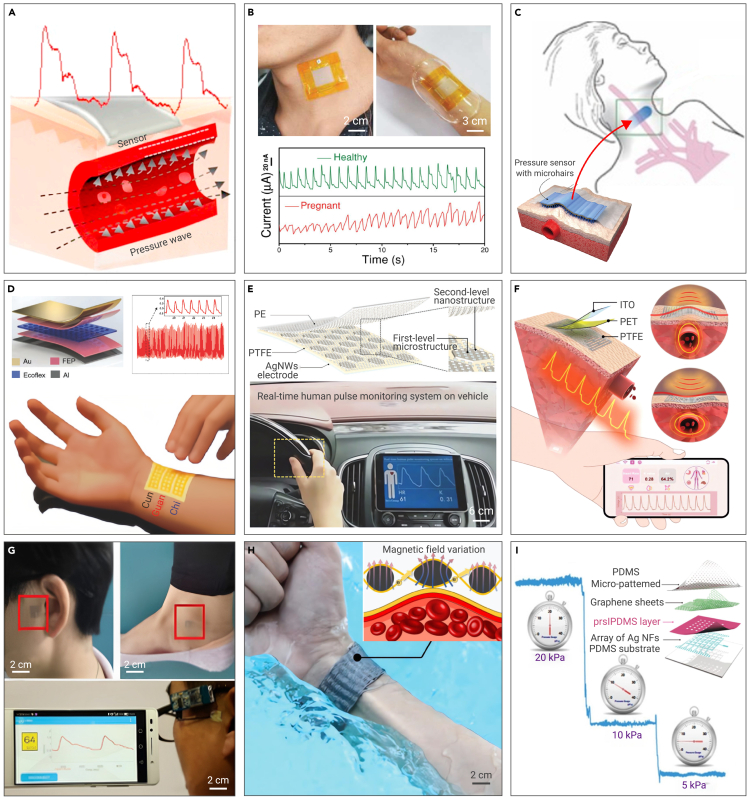
Flexible mechanical sensors for pulse and BP monitoring (A) Principle of flexible mechanical sensors for pulse and BP measurement.^60^ (B) Silk-micropatterned flexible piezoresistive sensor for wrist pulse.^61^ (C) Microhair flexible capacitive sensor for jugular vein pulse.^62^ (D) Sandwich flexible piezoelectric sensor for radial artery pulse.^63^ (E) Multilevel microstructured flexible piezoelectric sensor for fingertip pulse.^64^ (F) Kirigami-inspired flexible triboelectric sensor for artery pulse.^65^ (G) Woven sensors for continuous BP measurement of fingertip, wrist, ear, and ankle.^66^ (H) Flexible pressure sensor based on soft magnetoelastic fibers for underwater arterial pulse.^67^ (I) Stretchable pressure sensor arrays with microcage microstructure for different pulses under different external pressures.^68^

**Figure 3:**
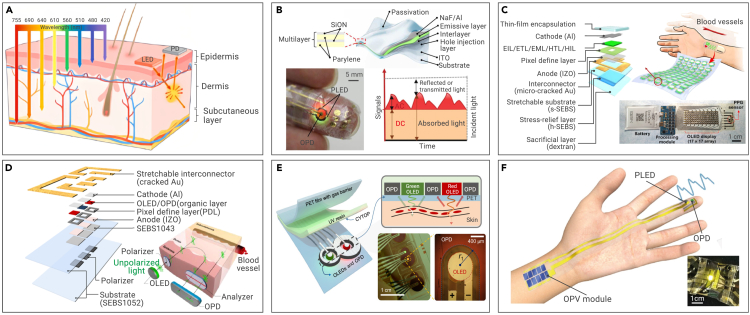
Flexible optical sensors for pulse and BP monitoring (A) Schematic of flexible optical sensors for biological monitoring.^69^ (B) Ultraflexible reflective pulse oximeter based on polymer LEDs.^70^ (C) A stretchable photonic patch for pulse and heart rate via OLEDs.^71^ (D) Stretchable optical sensor with an orthogonal polarizer-analyzer pair to overcome motion artifacts.^72^ (E) Ultralow-consumption pulse oximetry sensor.^73^ (F) Self-powered PPG sensor by combining air-operation-stable polymer LEDs, organic solar cells, and organic PDs.^58^

**Figure 4:**
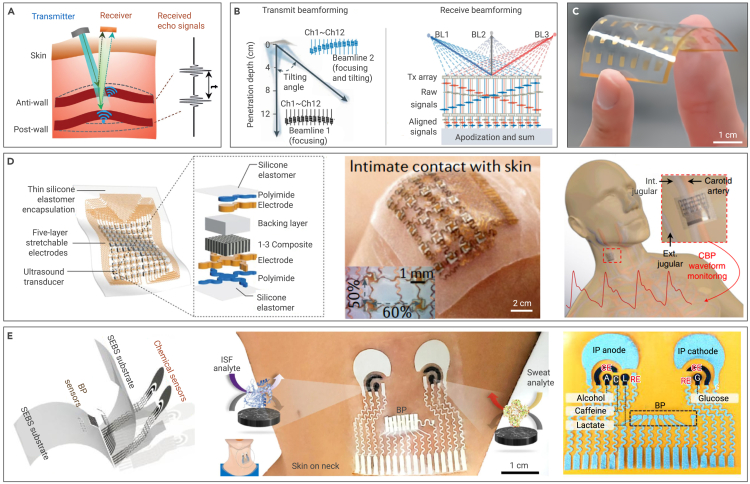
Flexible acoustic sensors for pulse and BP monitoring (A) The principle of flexible acoustic sensors for pulse and BP monitoring.^50^ (B) Transmission beamforming (left) and receiving beamforming (right) can substantially improve penetration depth and spatial resolution.^74^ (C) Optical image of a flexible capacitive micromachined ultrasound transducer.^75^ (D) Left: stretchable ultrasound array based on the “island-bridge” design. Center: intimate contact with nondevelopable surfaces. Right: stretchable acoustic sensor mounted on the human neck to monitor BP by capturing the pulsating vessel diameter of the carotid artery.^56^ (E) Left: the layer-by-layer layout of the integrated acoustic and electrochemical sensor. Center: illustrations of placement of the sensor and enzymatic chemical sensors for interstitial fluid and sweat. Right: simultaneous monitoring of BP along with sweat, alcohol, caffeine, and lactate and interstitial fluid glucose chemical markers.^76^

**Figure 5:**
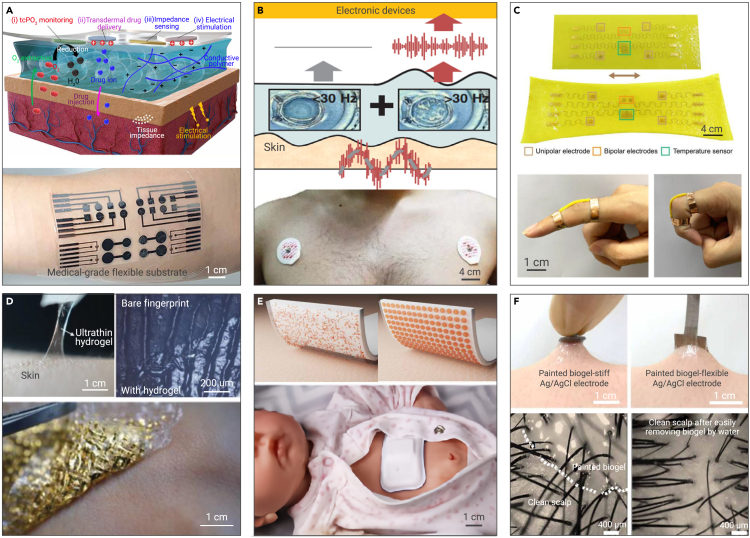
Flexible wet electronics for ECG monitoring (A) Hydrogel interface between human skin and wearable bioelectronics.^77^ (B) Hydrogel interpenetrated by chitosan and gelatin can eliminate dynamic noises under 30 Hz and filter biophysiological signals.^78^ (C) Multifunctional tendon-mimetic hydrogel without standing mechanics and functionality by anisotropically interpenetrating nanofiber composites.^79^ (D) Ultrathin hydrogel by a facile cold-lamination method, which is compliant with the glyphic lines and subtle minutiae on the skin without forming air gaps.^80^ (E) A thermally switchable adhesive silicone composite for neonatal patients enables fast, wirelessly triggered reductions in adhesive strength to eliminate the possibility of injury during removal.^81^ (F) A biocompatible on-skin paintable conductive biogel, which enables conformal contact and dynamic compliance with hairy skin.^82^

**Figure 6:**
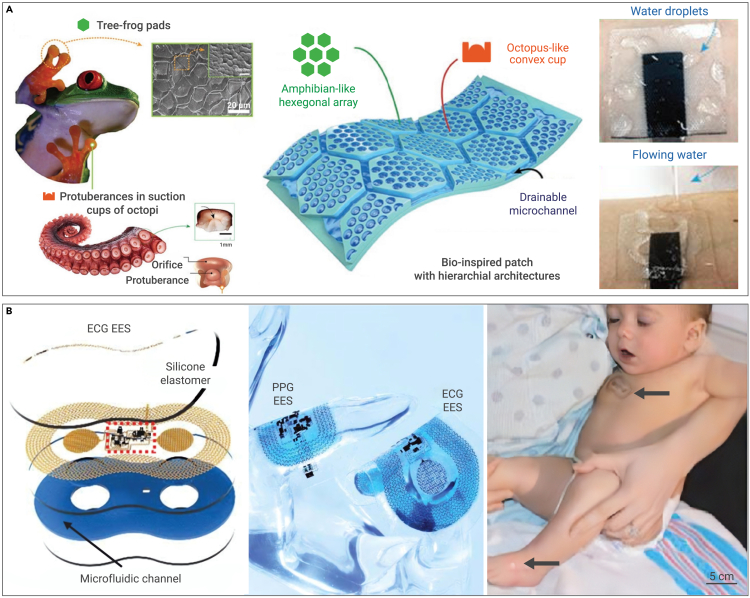
Flexible dry electronics for ECG monitoring (A) Highly air-permeable, water-drainable, and reusable patch bioinspired by tree frogs and octopi.^83^ (B) Ultrathin, ultrasoft, skin-like electrode for neonatal intensive care, which consists of filamentary metal mesh microstructures in fractal geometries.^57^

**Figure 7:**
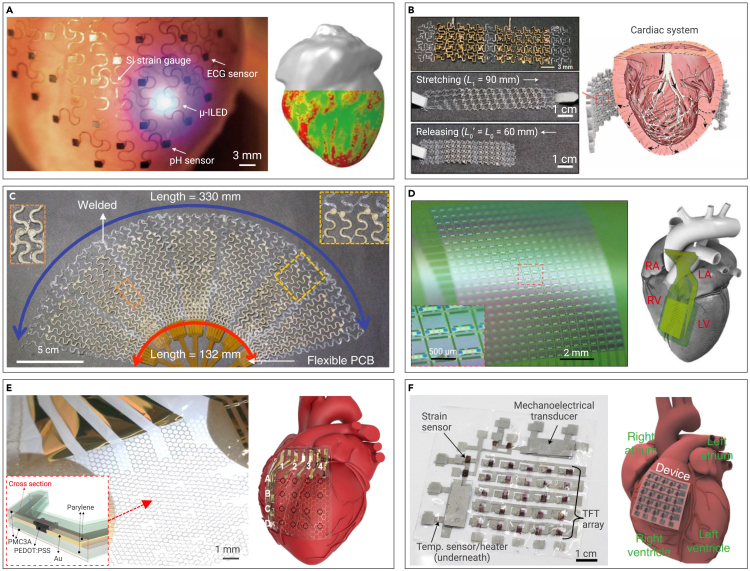
Implantable electrodes for ECG monitoring (A) 3D elastic membranes shaped precisely to match the epicardium of the heart by 3D printing.^84^ (B) Electromechanical cardioplasty using a wrapped elasto-conductive epicardial mesh.^85^ (C) Highly conductive, stretchable, and biocompatible electrodes by phase separation method.^86^ (D) Ultrathin, leakage-free, biocompatible dielectric layer that can completely seal the electrodes, allowing electrophysiological measurements via capacitive coupling between tissue and the electronics without the need for direct metal contact.^55^ (E) Active multielectrode array coated by soft poly(3-methoxypropyl acrylate) that can simultaneously achieve non-thrombogenicity, stretchability, and stability.^87^ (F) Epicardial bioelectronic patch that can perform spatiotemporal mapping of electrophysiological activity, strain and temperature sensing, and therapeutic capabilities (electrical pacing and thermal ablation).^88^

**Figure 8:**
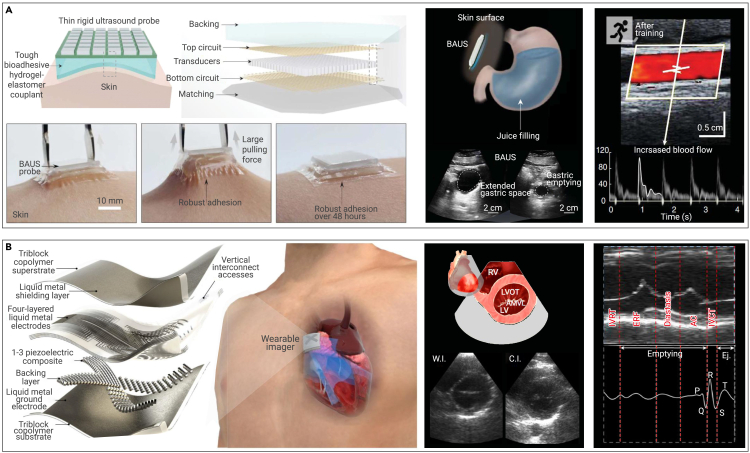
Flexible ultrasound imagers for echocardiography (A) A bioadhesive ultrasound imager for long-term continuous imaging of diverse organs.^89^ Left: a thin and rigid ultrasound probe, consisting of high-density and high-performance piezoelectric elements, is adhered to the skin via a bioadhesive couplant made of a soft, tough, and antidehydrating hydrogel-elastomer hybrid. Center: continuous imaging shows the dynamics of the stomach before and after drinking. Right: color-flow imaging of the carotid artery and diameter of the carotid artery. (B) A wearable cardiac ultrasound imager.^59^ Left: the flexible probe consists of piezoelectric transducer arrays, liquid-metal composite electrodes, and triblock copolymer encapsulation. Echocardiography from several views: B-mode image from a parasternal short-axis view (center) and M-mode images from a parasternal long-axis view (right).

**Figure 9:**
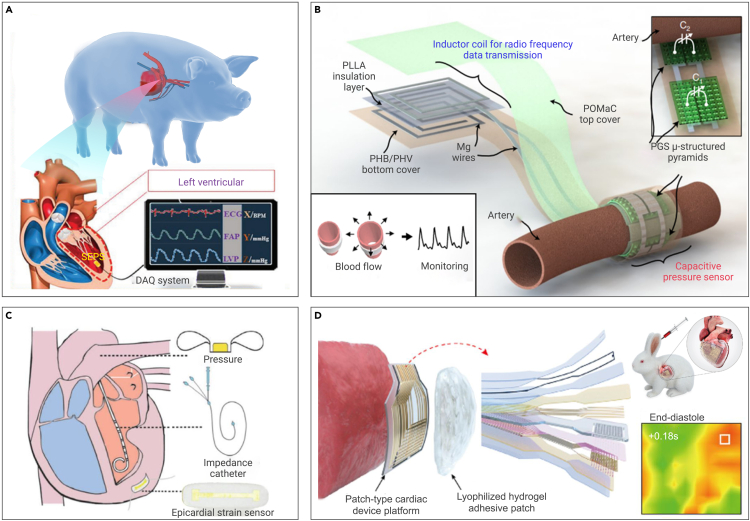
Flexible mechanical sensors for direct epicardium monitoring (A) Miniaturized, flexible, self-powered, ultrasensitive sensor for EP monitoring based on a TENG, which is integrated with a surgical catheter for minimally invasive implantation.^90^ (B) Biodegradable and flexible arterial pulse sensor for wireless monitoring of blood flow by wrapping around the artery.^91^ (C) Continuous heart volume monitoring after surgery by a fully implantable soft strain sensor made with biocompatible materials.^92^ (D) *In situ* diagnosis and simultaneous treatment of cardiac diseases using a single-device platform composed of an active-matrix, pressure-sensitive transistor array and biocompatible pacing electrodes with encapsulation layers.^93^

## Flexible electronics for pulse and BP monitoring

Among symptoms of CVDs, high BP has been shown to have the highest incidence.^94^ Monitoring arterial BP (ABP) is a vital and efficient means of detecting CVDs. Typically, BP is assessed through systolic BP (SBP) and diastolic BP (DBP).^95^^,^^96^ Traditional techniques for measuring BP primarily involve cuff-based methods, either the Korotkoff sound method or oscillometric method.^97^ An inflatable cuff is employed, with a stethoscope positioned over the brachial artery to capture the distinctive sounds of Korotkoff during cuff deflation. The SBP is estimated as the first sound detected, while the DBP is captured during the Korotkoff sound dissipation.^98^ Nevertheless, traditional cuff-based methods require a 3-min time gap between measurements, significantly hindering continuous BP measurement. Furthermore, although BP measurement with an arterial catheter is considered the gold standard for accuracy,^99^ its inherent invasiveness is unavoidable. Additionally, the use of a cuff exerts pressure on the arm or wrist, potentially leading to errors and discomfort. In light of these challenges, numerous studies have been conducted to explore alternative approaches for BP monitoring. The overarching goal is to identify and develop more continuous, user-friendly, and rapid devices. Flexible electronics are definitely promising candidates.^66^

### Flexible mechanical sensors for pulse and BP monitoring

Flexible mechanical electronics for pulse and BP monitoring are mainly flexible strain and pressure sensors. Based on the sensing mechanisms, flexible mechanical sensors can be classified into piezoresistive, capacitive, piezoelectric, and triboelectric devices.^100^^,^^101^^,^^102^^,^^103^ These sensors share similar configurations, featuring an active sensing component sandwiched by two electrodes. In response to strain or pressure, the active sensing components undergo mechanical changes and then lead to variations in resistance, capacitance, or electrical output. While piezoresistive and capacitive sensors necessitate external power sources, self-powered piezoelectric and triboelectric sensors can effectively transform pressure or strain into electrical signals.^104^^,^^105^^,^^106^ The active sensing components of piezoresistive sensors are typically composed of conductive networks, while the active sensing components of capacitive, piezoelectric, and triboelectric sensors usually are dielectrics.^107^^,^^108^ Utilizing these flexible pressure and strain sensors, the arterial pulse can be measured through local vibration of the radial or carotid artery via the tonometry method. This approach involves attaching sensors directly to the skin near the artery (Figure 2A).^60^

Flexible piezoresistive sensors that perform well in low-pressure regimens (<10 kPa) are frequently employed for pulse and BP monitoring. Figure 2B presents a silk-molded flexible piezoresistive sensor by Wang et al.^61^ which is capable of discriminating the pulse of a healthy person and a pregnant woman. To further improve the signal-to-noise ratio (SNR), Pang et al*.*^62^ proposed a flexible capacitive sensor for BP near the carotid artery, in which a compact microhair array is applied to the interface for signal amplification (Figure 2C). Flexible piezoelectric sensors are also common for pulse and BP monitoring because of their large but stable electromechanical coefficient. For example, Chu et al.^63^ constructed a sandwich-structured flexible piezoelectric sensor for radial arterial pulse measurement (Figure 2D). Wang et al.^64^ reported a multilevel microstructured flexible piezoelectric sensor for a tiny epidermal pulse like in the fingertips (Figure 2E). However, such sensors are generally prone to body motion artifacts, leading to inaccurate diagnosis and misinterpretation. This is mainly ascribed to weak adhesion or poor conformability, thereby causing an inconsistent interface between the devices and human skin. To eliminate motion artifacts, Meng et al.^65^ proposed a type of kirigami-inspired flexible triboelectric sensor for dynamic monitoring of the arterial pulse (Figure 2F).

Moreover, flexible mechanical sensors can be customized. For instance, patients may require pulse and BP measurements in particular areas of the skin, such as the ankles or temples. To meet this requirement, a woven sensor was developed for continuous measurement of fingertip, wrist, ear, and ankle (Figure 2G).^66^ The acquired data are transmitted wirelessly to personal mobile devices for ongoing BP monitoring. In practice, pulse and BP measurements are often subjected to external pressure. To address this issue, Zhao et al.^67^ proposed a flexible pressure sensor based on soft magnetoelastic fibers (Figure 2H), which is capable of measuring arterial pulse underwater without encapsulation. Zhang et al.^68^ demonstrated a type of stretchable pressure sensor array with a microcage microstructure, which can acquire clear pressure imaging and ultra-low cross-talk under varying external pressure (Figure 2I). Currently, the clinical application of flexible mechanical sensors for pulse and BP monitoring is primarily limited by (1) a lack of desired sensitivity required for accurate monitoring, (2) complex and time-consuming calibration that involves applying known pressure and comparing the output with a standard curve, (3) unreconciled trade-off between dimensions and functionality, (4) susceptibility to motion artifacts originating from unpredictable movement, (5) inadequate long-term stability and durability, (6) high power consumption, and so on. Future efforts need to first focus on sensitivity and sustainability while maintaining low energy consumption and ensuring consistent signal analysis.

### Flexible optical sensors for pulse and BP monitoring

Flexible optical sensors are another prevalent method to measure pulse and BP. Upon subjecting biological matter to light exposure, photons are absorbed and dispersed by cells and proteins, with penetration depth contingent upon wavelength. Reflected light from photophysical interaction with tissues imparts vital biometric information, including molecular content, morphology, and microstructure (Figure 3A).^69^ Flexible optical sensors utilized in cardiovascular monitoring commonly function in reflection mode. These sensors comprise a minimum of one pair of light-emitting diodes (LEDs) and photodetectors (PDs).^109^ LEDs serve as a light source that illuminates the skin, whereas PDs detect residual transmitted or reflected light. During the penetration process, blood and tissue consume a segment of the incident light, while the remaining light changes in intensity because of variations in blood volume in pulsating arteries during cardiac activity.^110^^,^^111^ The PDs then convert these intensity variations into electrical signals. Noteworthily, while the heart rate can be obtained from a single photoplethysmogram (PPG) signal, BP requires integration of PPG sensors with other bioelectric or mechanoelectrical sensors.^10^^,^^112^ It involves extracting BP-related parameters from multiple signals, such as pulse transit time and pulse arrival time.

When designing and producing flexible optical sensors, the selection of the light source is particularly important. Light sources may include lasers, LEDs, micro-LEDs (μLEDs), and organic LEDs (OLEDs) with different spectra and different biological effects. Monochromatic lasers in particular are capable of focusing on a single point, making them suitable for concentrated and powerful light exposure in specific areas.^113^ LEDs and μLEDs offer strong point illumination and are thus suitable for display and implantable electronics.^114^ Because OLEDs constitute thin-film devices using organic emitters, they can be employed as flexible and stretchable surface light sources, which can be used effectively for wearable devices. Quantum dot LEDs and perovskite LEDs provide exceptional chromatic purity, making them well suited for emitting light at a desired wavelength with a remarkably narrow spectrum.^115^ In Figure 3B, Yokota et al.^70^ demonstrated an ultra-flexible reflective pulse oximeter based on polymer LEDs, which can measure the blood oxygen concentration when laminated on a finger. Lee et al.^71^ realized a stretchable photonic patch for pulse and heart rate via OLEDs (Figure 3C).

To overcome motion artifacts, two orthogonal polarizers were employed to diminish the prevalence of scattered light from the epidermis, which is the main cause of motion artifacts (Figure 3D).^72^ The motion artifacts were suppressed more than 10-fold in comparison with those of rigid sensors. On the other hand, to realize long-term work, energy-efficient and self-powered methods were incorporated. Lee et al.^73^ devised a reflective patch-type pulse oximetry sensor with minimal power expenditure (Figure 3E). Jinno et al.^58^ developed a self-powered PPG sensor by combining air-operation-stable polymer LEDs, organic solar cells, and organic PDs (Figure 3F). By now, the clinical applications of flexible optical electronics are seriously constrained by the finite depth of light penetration within the body and energy consumption. Light with a wavelength of 400–500 nm can only go as deep as the epidermal layer, while light with a wavelength longer than 700 nm can reach deeper tissues beyond the dermis. However, deeper penetration often results in higher power consumption and more heat. In addition, flexible optical electronics for pulse and BP monitoring also suffer from (1) sensitivity to motion artifacts, (2) calibration and drift, (3) placement and fit, (4) the influence of ambient light, (5) limited measurement range, and so on. Future endeavors need to prioritize these concerns.

### Flexible acoustic sensors for pulse and BP monitoring

When sound travels in the body, it reflects, refracts, or scatters at the interface between different tissues or organs, causing pathological alterations influencing sound transmission.^56^^,^^116^ Compared with light and heat, sound can reach deeper positions. By scrutinizing the temporal span between the zenith of echo peaks originating from the anterior and posterior walls of an artery and combining this with the velocity of sound in biological tissues, the vascular caliber can be deduced (Figure 4A).^50^ This technique is especially suitable for monitoring the deep arteries because ultrasound beams are collimated and deeply penetrating. In conventional measurements, the pulse mode of a Doppler ultrasound machine is used, wherein a single ultrasound crystal emits and receives a sound beam (pulse model). Diverse samples can be employed to calculate information about heart or blood vessels (e.g., peak systolic velocity, end-diastolic velocity, and beat index).^117^ Thus, pulse waveforms can be obtained continuously. Furthermore, BP can be estimated based on the correlation between blood vessel diameter and BP.^50^ Operating at megahertz frequencies and wavelengths spanning hundreds of microns in tissues, ultrasound-based approaches enable the resolution of objects at the submillimeter scale.

Traditional rigid ultrasound probes often have limited acoustic coupling, but flexible ultrasound sensors effectively overcome this challenge. Flexible ultrasound sensors can be made of intrinsically flexible piezoelectric polymers or micromachined ultrasound transducers.^75^ A backing layer, typically made of metal-epoxy composites, absorbs excess ultrasound waves, dampens the ringing effect, and shortens the spatial pulse length, thereby improving spatial resolution in the axial direction.^118^ A matching layer, typically comprised of composites or metamaterials,^119^ may offset the mismatch of acoustic impedance between the device and the human body to enhance ultrasound transmission, thereby enhancing penetration depth. Generally, a single transducer only produces an exceedingly expansive beam and then leads to low spatial resolution. Alternatively, beamforming strategies using arrayed transducer elements are often employed. In transmit beamforming (Figure 4B, left), multiple elements can be programmed to synergistically transmit ultrasound waves.^74^ In-phase waves are superimposed to form a focal point with intensified acoustic energy. Therefore, a focused ultrasound beam enhances the spatial resolution and penetration depth of the device (Figure 4B, right).

Piezoelectric polymers exhibiting analogous acoustic impedances to human tissues possess an extensive bandwidth, which makes them outstanding ultrasound receivers (Figure 4C).^75^ In 2017, a flexible piezoelectric micromachined transducer array for ultrasound brain stimulation was proposed, marking its first application in structural health detection.^120^ The following year, a conformal ultrasound array was first shown in a clinical test, utilizing high-performance, rigid, 1–3 piezoelectric composites and an “island-bridge” layout (Figure 4D, left).^56^ With a penetration depth of 4 cm, the device has a mere 240-μm overall thickness. The serpentine conductive network offers greater than 50% biaxial stretchability with minimal impact on transducer performance, ensuring intimate adherence to nondevelopable surfaces (Figure 4D, center). The final wearable device enables noninvasive, continuous, and accurate monitoring of cardiovascular events from multiple body locations (Figure 4D, right). What’s more, flexible acoustic sensors have been combined with electrochemical sensors to monitor the signals of BP and multiple biomarkers without cross-talk between the individual sensors (Figure 4E, left),^76^ such as glucose in interstitial fluid and lactate (Figure 4E, center) and caffeine and alcohol in sweat (Figure 4E, right). Presently, the primary challenge for clinical application of flexible acoustic sensors lies in accuracy since such devices are susceptible to external environmental noise, such as background sounds or vibrations. Similar to the flexible mechanical and optical counterparts discussed above, flexible acoustic sensors also have issues like motion artifacts, calibration, and power consumption. Researchers and engineers are actively addressing these limitations by developing advanced signal-processing algorithms, optimizing sensor designs, improving calibration methods, and exploring novel materials and fabrication techniques.

## Flexible electronics for ECG monitoring

ECG is a pivotal diagnostic examination that measures and records cardiac electrical impulses via an electrode pair mounted on the chest. Deciphering ECG enables medical practitioners to check for an array of cardiac afflictions, including arrhythmias, myocardial infarction, structural anomalies, and sundry cardiovascular maladies.^121^ This noninvasive and economical approach serves as a foundation for diagnosis, surveillance, and management of diverse cardiac conditions, facilitating prompt and judicious intervention, promoting patient prognosis, and mitigating complications concomitant with cardiac diseases. Contemporary medicine ubiquitously employs ECG in routine evaluations, emergency scenarios, and preoperative assessment. Typically, ECG contains tiny adhesive electrodes attached to the limbs and chest, which are subsequently connected to an ECG recording apparatus.^122^ In contrast to conventional ECG methodologies that constrain the patient’s movement, flexible electronics are able to adapt to complex skin surfaces and thus provide sufficient comfort. The integrated system coupled with the electrodes has independent ability to perceive and process the data for diagnosis, liberating people from the traditional heavy equipment and leaving them free to continue their daily life during measurement. Moreover, it is cost-effective and disposable. Until now, flexible electronics for ECG monitoring are primarily flexible mechanical electronics, which are further divided into epidermal and implantable electrodes based on their application scenarios.

### Epidermal electrodes for ECG monitoring

Epidermal ECG typically contains two kinds of configurations, flexible dry and wet electrodes.^123^ Dry electrodes are engineered to obtain electrical signals from the body without the need for gel or paste. These electrodes typically incorporate a conductive medium on the electrode surface, which can directly build up the current with the integument.^124^ Wet electrodes generally provide superior signal quality because of low impedance, especially when capturing ECG signals with heightened precision.^125^^,^^126^^,^^127^^,^^128^ Nevertheless, the long preparation process for the gel or paste is laborious and may cause discomfort or cutaneous irritation for particular individuals. Moreover, wet electrodes leave residues that necessitate post-procedure cleaning. Comparatively, dry electrodes are more facile to apply and offer excellent convenience for the patient.

Flexible wet electrodes frequently feature ultra-flexible, gas-permeable, and low-impedance hydrogels containing a porous matrix immersed in a certain percentage of water.^129^^,^^130^ The low Young’s modulus and hydrated feature facilitate exceptionally conformal contact with the skin. The ultra-thin configuration of these flexible electrodes diminishes stress concentration, preventing mechanical failure upon body motion. Nevertheless, hydrogels are facing two challenges: first, water loss through evaporation, which not only contaminates circuits but also weakens mechanical properties, and second, functionalization without sacrificing the original characteristics.^131^ To address the former issue, hydrogels are encapsulated in water-resistant elastomers, and highly hygroscopic LiCl salt is integrated into the hydrogel.^132^ To tackle the latter problem, numerous studies have altered the composition and structure of the cross-linked polymer network within hydrogels.^133^ However, because hydrogels weaken the signal acquisition capacity of wet electrodes, the signal transmission intensity and precision of wet electrodes should be enhanced prior to functionalization.

Ultra-thin, functionalized hydrogels with exceptional gas permeability and minimal impedance have been proposed to establish a harmonious interface between wearable bioelectronics and human skin (Figure 5A).^77^ The porous framework and wafer-thin dimensions accelerate transit of target molecules across the interface and thus optimize signal acquisition fidelity. Furthermore, hydrogels incorporated with chitosan and gelatin have the capacity to ignore dynamic disturbances below 30 Hz, which serves as an adaptable pass filter that ensures acquisition of high-quality signals from patients while minimizing signal processing for advanced bioelectronics (Figure 5B).^78^ Recently, Sun et al.^79^ devised a class of multifunctional tendon-mimetic hydrogels exhibiting unyielding mechanics and functionality via anisotropically interpenetrating nanofiber composites (Figure 5C). The flexible strain sensor based on an anisotropic composite hydrogel is capable of characterizing the motion of a joint across various amplitudes. Cheng et al.^80^ unveiled an ultrathin hydrogel by a facile cold lamination technique, yielding compliance with glyphic lines and subtle minutiae on the skin without engendering air gaps (Figure 5D). On the other hand, with clinical applications in mind, Jinkins et al.^81^ provided a material strategy that can realize rapid, wirelessly actuated reductions in adhesive strength, thereby precluding injury during removal, which is a crucial concern for neonatal and pediatric patients (Figure 5E). Wang et al.^82^ introduced a biocompatible, paintable, and conductive on-skin biogel that ensures conformal contact and dynamic compliance with hairy skin (Figure 5F).

Dry electrodes for ECG have several benefits over their wet counterparts: (1) convenience and hygiene because there is no need for a gel or paste, (2) enhanced comfort because of less irritating designs, and (3) durability, mobility, and portability. Intimate dermal adhesion is a fundamental requirement for achieving optimal performance with dry electrodes. Bionics is proven to be a powerful and effective approach in this regard. Historically, a myriad of ingenious concepts has been motivated by biological systems and evolutionary refinement transpiring over eons. Bioinspired dry electrodes have been extensively examined. For instance, inspired by the microchannel networks of tree frog toe pads and the convex cups of octopus suckers, Kim et al.^83^ proposed a highly breathable, water-drainable, and reusable patch, greatly improving adhesion and omnidirectional peel resistance (Figure 6A). Similarly, inspired by the corrugated suction cups of male diving beetle forelegs, Min et al.^134^ devised an electro-adhesive patch that exhibits remarkable wet adhesion enhancement, sweat drainability, and highly electrical stability under tensile strain in both dry and wet conditions. Besides, material engineering and structural engineering are feasible methods to enhance the adhesion of dry electrodes. Yan et al.^135^ formed approximately 10-nm-thick freestanding sheets by spin-coating films containing flakes of semiconducting material, by which stretchable, adaptable, and breathable van der Waals thin electrodes were realized. Chung et al.^57^ introduced an ultra-thin, ultra-soft, skin-like electrode for neonatal intensive care, comprising filamentary metal mesh microstructures in fractal geometries (Figure 6B). Noteworthily, artificial intelligence can also greatly enhance the measurement accuracy and range of such electrodes. For example, Zhuang et al.^136^ introduced a very interesting paradigm that realized facial expression recognition via deep-learning-assisted epidermal electrodes.

### Implantable electrodes for ECG monitoring

Beyond conventional *in vitro* assessment via epidermal electrodes, ECG can also be obtained through implantable cardiac patches that are directly integrated into the heart’s surface. The great advantage is that these patches can provide supplemental monitoring and treatment for CVDs, broadening the utilization scope of epidermal electrodes. For example, many arrhythmias (e.g., atrial fibrillation and ventricular tachycardia) originate from endocardial substrates.^137^ Consequently, delineating arrhythmogenic activity in specific cardiac regions is crucial for devising definitive therapies, such as cardiac ablation.^138^ Recent advancements in materials and mechanics for flexible electronics present a promising opportunity to overcome this challenge, providing high spatial resolution and intimate mechanical integration with myocardial tissue with negligible impact on natural motion. This is of immense clinical significance.^139^ Moreover, another primary objective of implantable cardiac patches is restoration or enhancement of cardiac function. These patches may have the potential to strengthen weakened heart muscle, augment contractility, and potentially optimize blood flow, which is significant for patients afflicted with conditions such as myocardial infarction (heart attack) or heart failure.

The essential challenge is to make implantable patches sufficiently conformable to fully wrap rugged, time-dynamic surfaces of the heart. Kim et al.^139^ demonstrated a type of electronic sensing and actuating network for expansive, intricate-geometry cardiac mapping and treatment, which is capable of conformally encasing the epicardium while maintaining steadfast contact without sutures, mechanical fixtures, tapes, or surgical adhesives. Similarly, Xu et al.^84^ crafted three-dimensional pliant membranes to correspond with the epicardium via 3D printing, providing a platform for deformable arrays of multifunctional sensors, electrodes, and optoelectronic components (Figure 7A). In a comparable manner, Park et al.^85^ utilized 3D printing to construct an epicardial mesh comprised of electrically conductive and mechanically elastic material, facilitating electromechanical cardioplasty by emulating innate cardiac tissue and bestowing cardiac conduction system functionality (Figure 7B). Nevertheless, the implantable patches produced through 3D printing make a trade-off between conductivity and elasticity. As an alternative, Choi et al.^86^ reported an Ag-Au nanocomposite consisting of ultra-long gold-coated silver nanowires embedded in an elastomeric block-copolymer matrix via a phase separation technique, which can concurrently satisfy conductive, stretchable, and biocompatible prerequisites (Figure 7C).

To address the risk of biological fluid infiltration into the underlying electronics, Fang et al.^55^ demonstrated an ultra-thin, leak-proof, biocompatible dielectric layer capable of hermetically sealing electrodes, facilitating electrophysiological measurements via capacitive coupling between tissue and electronics without direct metal contact (Figure 7D). To further enhance stretchability and conformability, Lee et al.^87^ devised an active multi-electrode array enveloped in a flexible poly(3-methoxypropyl acrylate) coating, concurrently achieving non-thrombogenicity, stretchability, and stability (Figure 7E). To enrich functionality, Sim et al.^88^ presented an epicardial bioelectronic patch that is capable of executing spatiotemporal mapping of electrophysiological activity, as well as strain and temperature sensing. Furthermore, it enables therapeutic capabilities (electrical pacing and thermal ablation) and self-powering from heartbeats (Figure 7F).

Implantable electrodes can furnish more specific information regarding cardiac signals and can also be employed for complex treatment of patients with severe cardiovascular blockages. However, the trade-off between conductivity and stretchability necessitates continued exploration of novel materials and structures. Although the current bioelectronic patches are primarily intended for temporary epicardial implantation, incorporating anti-inflammatory agents and materials, coupled with a detailed examination of induced fibrosis, could potentially pave the way for chronic implant development. Among the electrodes employed for ECG measurement, wet electrodes are dominant at this stage. The related research mainly focuses on the lightweight design and functionalization of hydrogels. Although dry electrodes have the advantage of stronger signals, they are prone to artifacts because of insufficient adhesion to the skin. Current research of dry electrodes concentrates on enhancing the interface between the electrodes and skin to improve the level of stability.

## Flexible electronics for echocardiogram monitoring

Echocardiography is a noninvasive imaging technique used to assess the structure and function of the heart. It utilizes ultrasound waves to produce real-time images of the heart and its various components. Echocardiography has evolved into a customary practice for diagnosing, managing, and monitoring individuals with suspected or confirmed cardiovascular disorders. This method is one of the most extensively employed diagnostic imaging modalities in the field of cardiology. It offers an abundance of valuable insights, including the heart’s dimensions and configuration (quantification of internal chamber size), functional capacity, localization and magnitude of any tissue impairment, and appraisal of valvular components. The distinct advantages of echocardiography lie in its noninvasive and real-time nature devoid of any noticeable risks or side effects. Echocardiograms not only generate ultrasound depictions of cardiac structures but also facilitate precise evaluation of blood circulation in the heart through Doppler echocardiography, employing pulse wave (PW) and continuous wave (CW) Doppler techniques.^140^^,^^141^ In conventional echocardiography measurements, the patient is positioned appropriately, usually lying on the left side or back. A coupling agent is applied to the chest surface to enhance sound wave transmission, and then a rigid probe is placed on the skin to scan different sections. Such a procedure has certain limitations, like operator dependency, interobserver and intraobserver variability, limited accessibility because of the bulky and specialized equipment, the need for post-procedure cleaning, and requirement of patient posture. Flexible electronics that are available as wearable, implantable, or highly integrated configurations enable continuous and real-time monitoring of echocardiography data, opening a new way to circumvent the conventional medical workflow.

The paramount challenges of flexible ultrasound electronics for high-performance cardiac imaging come from two aspects: generating ultrasound Doppler effects and confirming the Doppler angle *θ*. To remove the former obstacle, an angled transducer array is used to produce tilted ultrasound beams, thus ensuring relative motion between the beams and scatterers. The latter issue is resolved by employing piezoelectric transducer arrays with varying inclination angles, thus negating the Doppler angle’s impact on velocity measurements. Grounded in these principles, Wang et al.^142^ devised a flexible, phased-array ultrasound electronic system that is capable of monitoring hemodynamic signals from tissues up to 14 cm beneath the skin. Active focusing and steering of ultrasound beams across a spectrum of incident angles guarantee the precision of target regions. Nevertheless, despite the improved wearability afforded by piezoelectric elements on a stretchable substrate, limitations still exist, including (1) diminished imaging resolution because of low-density elements, (2) inconsistent image quality caused by unpredictable deformation, and (3) device failure arising from the inherent frailty of current stretchable materials.

Regarding the aforementioned challenges, a pair of distinct solutions have been proposed: a rigid ultrasound imager affixed by bioadhesive couplant^89^ and an intrinsically stretchable ultrasound imager.^59^ The initial proposition, conceived by Wang et al.,^89^ emphasizes the adhesive couplant over the ultrasound imager. A thin and rigid ultrasound probe consisting of high-density, high-performance piezoelectric components is adhered to the skin via a bioadhesive couplant made of a soft, robust, and antidehydrating hydrogel-elastomer amalgam (Figure 8A, left).^89^ This couplant facilitates ultrasound penetration, insulates the rigid probe from skin distortion, and ensures comfortable adhesion for over 48 hours. Consecutive imaging of the stomach (Figure 8A, center) and carotid artery (Figure 8A, right) substantiates the feasibility and efficacy of this technique. The latter approach, proposed by Hu et al.^59^ concentrates on enhancing the stretchability and adaptability of the ultrasound probe through innovations of device design and material fabrication. The flexible probe is composed of piezoelectric transducer arrays, liquid metal composite electrodes, and triblock copolymer encapsulation (Figure 8B, left).^59^ Acquired echocardiography from multiple perspectives, including B-mode images from parasternal short-axis views (Figure 8B, center) and M-mode images from parasternal long-axis views (Figure 8B, right), demonstrates that such a flexible probe significantly improves imaging precision. This advancement is of paramount significance for dynamic wearable devices of cardiac performance. Comparing the two methodologies, it becomes evident that achieving both functionality and convenience is crucial for flexible ultrasound electronics. However, these attributes are presently incompatible. Therefore, the optimization of rigid probes from the aspects of comfort and enhancement of soft probes from the aspects of imaging performance present both logical opportunities and challenges.

## Flexible electronics for direct epicardium monitoring

Direct epicardium monitoring refers to directly measuring or monitoring the electrical activity, blood flow, or other physiological parameters of the epicardium—the outer layer of the heart.^143^^,^^144^ Compared with noninvasive echocardiography, direct epicardium monitoring allows healthcare professionals to gather precise and real-time information about the heart’s functions and conditions, such as accurate assessment of heart rhythm, evaluation of ischemic heart disease, and assessment of epicardial substrate.^145^ It can help guide surgical decision-making, identify and treat arrhythmias, evaluate myocardial ischemia, and monitor the effects of interventions in real time. Moreover, direct epicardium monitoring is crucial for studying the physiological and pathological processes that occur in the heart.^146^ By directly measuring epicardial electrical activity, researchers can gain insights into the mechanisms of arrhythmias, myocardial ischemia, and other heart conditions.^147^^,^^148^ This knowledge can drive advancements in treatment strategies and device development. However, as an invasive strategy, direct epicardium monitoring has certain risks, including infection, bleeding, and damage to the heart tissue.^149^^,^^150^ Flexible electronics that are biocompatible, biodegradable, wireless, self-powered, and ultrathin but multifunctional offer potential for minimizing invasive procedures.^151^ In Subsection “implantable electrodes for ECG monitoring”, flexible implantable electrodes for ECG monitoring have been shown, here flexible implantable sensors for other purposes are further demonstrated.

Realization of direct epicardium monitoring mainly relies on flexible mechanical sensors.^152^ Typically, those sensors encircle arteries or adhere to the myocardial surface. For example, because of blood flow pulsation, the vessel diameter change of the artery over time is measurable via a capacitive sensor around the artery. Shifts of capacitance affect the resonant frequency of the inductor-to-capacitance-resistance (LCR) circuit, which can be observed wirelessly through the skin in a battery-free manner by utilizing inductive coupling to an external reader coil. Flexible mechanical sensors based on triboelectric nanogenerators (TENGs) are able to evaluate endocardial pressure and convert energy from blood flow in the heart’s chambers into electricity.^153^ The electrical output of the device can reflect physiological and pathological cardiovascular states, including endocardial pressure (EP), ventricular fibrillation, and premature ventricular beats.^144^ Moreover, flexible mechanical sensors can be directly affixed to the myocardial surface for *in situ* cardiac data acquisition.^154^

A crucial indicator of the heart’s ability to pump blood and the effects of therapeutic approaches is EP, such as atrial and ventricular pressure. In clinical practice, intermittent EP measurement often unwittingly neglects transient/silent symptoms, which will result in misdiagnosis of patients. Cardiac catheterization is currently the sole effective method for obtaining EP data in a clinical setting, but it is costly, invasive, and impractical for long-term and continuous data collection. In addition, an external bulky recorder connected with cardiac catheterization also has a series of shortcomings, such as complex operations and inadequate patient compliance. Alternatively, flexible mechanical sensors based on TENGs can be attached to the heart and continuously capture EP information. Moreover, they can be coupled with a surgical catheter, minimizing invasive implantation (Figure 9A).^90^ In a porcine model, the sensor is implanted into the left ventricle and left atrium, which demonstrates an admirable response in low- and high-pressure environments.

Continuous monitoring of vessels would be valuable to prevent potential tissue/graft loss both immediately after surgery and following patient discharge. Although existing methods can provide accurate and valuable insights, they are costly and complex, necessitating highly trained clinicians for operation. In addition, none of these methods address the need of monitoring patients who have been discharged from the hospital. Previous flexible mechanical sensors detect changes in blood flow velocity by detecting variations in blood vessel diameter. A cuff-type sensor has proven to be the most suitable choice for encircling arteries. However, the process of wrapping the arteries is cumbersome, and subsequent removal of sensors makes them prone to secondary injury or infection. Therefore, degradable materials are more suitable for making such sensors. Boutry et al.^91^ developed a flexible pressure sensor comprised entirely of biodegradable materials, which is capable of gauging arterial blood flow (Figure 9B).

In other cases, critically ill patients are prone to vascular paraplegia after surgery, which correlates with peripheral blood volume leakage that constrains the heart’s capacity to pump blood with each contraction (stroke volume, cardiac output).^155^^,^^156^ Echocardiography is the prevailing method for assessing cardiac volume in clinical practice. However, it can only yield a single volume value per measurement. A continuous and more reproducible method for heart volume measurement entails intra-cardiac electric impedance by utilizing a catheter positioned in the heart. Dual et al.^92^ continuously measured left heart volume by using piezoresistive strain sensors composed of biocompatible materials and placed directly on the heart’s exterior surface (Figure 9C). The flexible mechanical sensor is made of gold-coated TiO_2_ nanowires embedded in a soft polydimethylsiloxane (PDMS) elastomer, which can fit smoothly into the heart wall without impeding cardiac contraction. Furthermore, flexible mechanical sensors can be assembled as platforms (array of pressure-sensitive transistors, low-impedance electrodes, and alginate-based hydrogel) and attached to the heart to monitor the temporal and spatial distribution of cardiac pressure during its movement, including color mapping and traces of EP and other physiological characteristics (Figure 9D).^93^

Implantable mechanical sensors are more suitable for arrhythmia, post-surgical vascular paraplegia, and related disorders, which can directly and continuously monitor EP and vascular velocity. Nevertheless, their sensitivity remains at a low level. Due to material and design limitations, enhancements in size and biocompatibility are necessary for these implantable sensors. Moreover, depending on the host organ and surrounding tissue, the unintentional load on the sensor can change the sensor’s behaviors and lose its original position, leading to its failure. To avoid this situation, a thorough calibration process and data analysis procedure must be developed to eliminate potential drift.

## Conclusion and outlook

Flexible electronics have the potential to revolutionize cardiovascular healthcare monitoring by enabling innovative and patient-centric approaches to diagnosis, monitoring, and treatment. However, many capabilities and device prototypes in this domain are still in infancy, predominantly confined to laboratory investigation or demonstrated exclusively under optimal conditions without consideration of the clinical environment. Unlike numerous previous reviews of flexible electronics, which primarily focus on concepts, materials, functions, designs, or fabrication, this review aims to arouse the interest of researchers in related fields and inspire their curiosity from a perspective of pragmatic clinical application. Toward this end, we try to answer the following questions: What advantages can flexible electronics provide for cardiovascular healthcare monitoring? What are the current limitations? How can we get qualified flexible electronics for clinical applications? On one hand, this review serves as a kind of layman’s review that introduces flexible electronics to researchers and practitioners from other fields, especially from clinical medicine. On the other hand, this review shows more possibilities and challenges of clinical requirements to the specialists in flexible electronics. Finally, we close this discussion by identifying several important branches for future work.

First, materials play a crucial role in the design, fabrication, and performance of flexible electronics. Their selection depends on several factors, including the desired mechanical and electrical properties, device functions, and manufacturing processes. Continuous exploration and development of new materials or modification of existing ones to improve the flexibility, durability, and performance of flexible electronics is the route one must take. Implantable flexible electronics should also consider biocompatibility. Second, more advanced mechanical designs are needed to reconcile the ever-present tradeoff between high mechanical properties (e.g., stretchability and conformability) and high electrical performance (e.g., conductivity and stability). Newly popular flexible mechanical metamaterials, such as kirigami, origami, and topological mechanical metamaterials, deserve attention and research. Third, acquiring more precise cardiovascular information is essential. Flexible electronics need more advanced designs to improve the intensity of the information. Fourth, most flexible electronics focus on their own functions and still rely on wired, external, and extensive data acquisition systems. Future attention should be paid to wireless transmission, miniaturization, and integration. Fifth, flexible electronics have the potential to be applied to other cardiovascular healthcare monitoring methods, such as computed tomography and magnetic resonance imaging. Last but not least, electrical risks (e.g., short-circuiting and overheating), mechanical risks (e.g., fracture or delamination and mechanical wear), and biological risks (e.g., biocompatibility and infection risk) associated with use of flexible electronics in cardiovascular healthcare need special attention. Strategies that can withstand mechanical stress, prevent short-circuits, and promote tissue integration are waiting to be explored. Rigorous testing procedures and regulatory standards should be in place to ensure the safety and effectiveness of these devices before they are used in clinical settings.

Except for the self-improvement of flexible electronics, the rapid development of artificial intelligence is playing an increasingly significant role in cardiovascular healthcare. Advanced algorithms are able to greatly accelerate data processing, shorten the reaction time, weaken motion artifacts, lower physician dependency, enhance clinical decision-making, improve patient outcomes, and advance cardiovascular care. Flexible electronics are an almost ideal platform for implementing artificial intelligence (AI)-assisted diagnosis, with vast amounts of data being offered to machine learning algorithms and training datasets. The prospect of developing an AI-assisted healthcare system like GPT-4 is truly exciting. Finally, flexible electronics are a highly interdisciplinary area of research, exhibiting inherent complexity and diversity. Successful development of these devices relies on universal collaboration among researchers and practitioners from a wide array of disciplines, such as materials science, chemistry, physics, mechanics, algorithm and software development, hardware design, clinical medicine, and beyond. Nonetheless, the potential of flexible electronics to enhance patient care, customize patient diagnosis and treatment, and improve the accuracy of outcomes makes them an area of active exploration and innovation.

## Lead contact website

Shan Jiang: https://hz.xidian.edu.cn/info/1137/4374.htm.

Yuan Huang: https://orcid.org/0000-0002-1420-8932.

Shoujun Li: https://www.fuwai.com/Hospitals/Doctors/Index/194.
